# Variations in Marine Bacterial and Archaeal Communities during an *Ulva prolifera* Green Tide in Coastal Qingdao Areas

**DOI:** 10.3390/microorganisms10061204

**Published:** 2022-06-13

**Authors:** Guihua Zhao, Hui He, Hualong Wang, Yantao Liang, Cui Guo, Hongbing Shao, Yong Jiang, Min Wang

**Affiliations:** 1 College of Marine Life Sciences, Institute of Evolution and Marine Biodiversity, Frontiers Science Center for Deep Ocean Multispheres and Earth System, Ocean University of China, Qingdao 266003, China; 11190611056@stu.ouc.edu.cn (G.Z.); wanghualong@ouc.edu.cn (H.W.); liangyantao@ouc.edu.cn (Y.L.); guocui@ouc.edu.cn (C.G.); hbshao@ouc.edu.cn (H.S.); yongjiang@ouc.edu.cn (Y.J.); 2 The Affiliated Hospital of Qingdao University, Qingdao 266000, China; 3 OUC-UMT Joint Academic Centre for Marine Studies, Qingdao 266003, China

**Keywords:** *Ulva prolifera* green tide, bacteria, archaea, co-occurrence network, Illumina high-throughput sequencing

## Abstract

Green tides caused by *Ulva prolifera* occur annually in the Yellow Sea, potentially influencing the marine microorganisms. Here, we focused on the variations in marine bacterial and archaeal communities during an *U. prolifera* green tide in coastal Qingdao areas with Illumina high-throughput sequencing analysis. Our results revealed that the diversity and structure of bacterial and archaeal communities, as well as the organization and structure of microbial co-occurrence networks, varied during the green tide. The decline phase may be favorable to the bacterial and archaeal diversity and richness. The bacterial community, as well as the archaeal community, showed clear variations between the outbreak and decline phases. A simpler and less connected microbial co-occurrence network was observed during the outbreak phase compared with the decline phase. *Flavobacteriales* and *Rhodobacterales* separately dominated the bacterial community during the outbreak and decline phase, and *Marine Group II* (*MGII*) dominated the archaeal community during the green tide. Combined with microbial co-occurrence network analysis, *Flavobacteriales*, *Rhodobacterales* and *MGII* may be important organisms during the green tide. Temperature, chlorophyll *a* content and salinity may have an important impact on the variations in bacterial and archaeal communities during the green tide.

## 1. Introduction

Green tide is a kind of ecological disaster caused by macroalgae mainly including genera *Ulva*, *Chaetomorpha* and *Cladophora*. Since 2007, green tide primarily driven by *Ulva prolifera* has occurred annually in the Yellow Sea, which is regarded as the largest green tide in the world [1,2]. *U. prolifera* generally floats on the surface of seawater, posing a serious threat to coastal tourism, fishery and environment [3,4]. The outbreak of *U. prolifera* green tide affects not only the nutrient contents of seawater but also the interactions among marine organisms, and further influences the marine biogeochemical cycles [5,6,7,8]. During the decomposing process of *U. prolifera*, it can consume dissolved oxygen resulting in water hypoxia or even anoxia, and also release hydrogen sulfide into seawater, which is hazardous to marine organisms [9,10]. In addition, decomposing *U. prolifera* can release nutrients into seawater, causing a brief eutrophication and possibly leading to secondary disasters such as jellyfish blooms [6,11].

Due to extremely high diversity and various ecological functions, microorganisms that are involved in biogeochemical cycles, primary production and microbial loop are the keystone of marine ecosystems [12,13,14]. Microorganisms, on the other hand, are regarded as sensitive indicators in characterizing the environmental changes [15]. Algal blooms have crucial effects on the microbial community that may differ depending on causative species, bloom phases and environmental factors [16,17]. For example, *Actinobacteria* and *Betaproteobacteria* dominated the bacterial community during a cyanobacteria bloom, while *Flavobacteria* and *Alphaproteobacteria* were the major taxa during a diatom bloom [16,18]. Zhou et al. [19] reported that *Gammaproteobacteria* and *Bacteroidetes* dominated the bacterial community during the initial phase of a dinoflagellate bloom, while *Alphaproteobacteria*, *Cyanobacteria* and *Actinobacteria* were the major bacterial taxa during the onset and termination phases. Variations in the archaeal community also occurred during a dinoflagellate bloom, with methanogens and ammonia-oxidizing archaea being the main taxa during the early and late phases, respectively [19].

It is reported that *U. prolifera* may settle in seawater southeast of the Shandong Peninsula, particularly along the coastal Qingdao areas [20]. Variations in bacterial community during the *U. prolifera* green tide have been reported successively [8,21,22,23,24,25]. For example, Zhang et al. [21] found that the bacterial community varied during an *U. prolifera* green tide, and the abundance of nitrogen-fixing bacteria dramatically increased during the outbreak phase. *U. prolifera* green tide in coastal Qingdao areas led to a reduction in bacterial diversity and abundance, and some functional groups, such as sulfate-reducing bacteria and *Cytophaga*-*Flavobacter*-*Bacteroides*, were also affected by the *U. prolifera* green tide [8]. In recent years, microbial interactions have been considered to be vital drivers in determining the algal blooms [26]. Co-occurrence network analysis has been used in a variety of environments including seawater, sediments and soils [27,28,29], and is an effective tool for the reflection of interactions between microorganisms, which is critical to the assembly and stability of microbial community [30,31,32]. By means of co-occurrence network analysis, Zhou et al. [33] found that time-shifted interdependencies were prevalent in the bacterioplankton community, and *Rickettsiaceae*, *Saprospriaceae*, *Winogradskyella*, *Flavobacteriaceae*, *Chitinophagaceae* and *NS 11–12* were the core taxa during a dinoflagellate bloom. However, to our knowledge, microbial interactions during an *U. prolifera* green tide derived from the co-occurrence network analysis have not been reported yet. In this study, variations in marine bacterial and archaeal communities during an *U. prolifera* green tide in coastal Qingdao areas were explored through the Illumina high-throughput sequencing method based on the 16S rRNA gene. Our primary goals were: (i) to explore the variations in bacterial and archaeal communities during an *U. prolifera* green tide, (ii) to identify the shift of microbial interactions using co-occurrence network analysis during an *U. prolifera* green tide, and (iii) to discuss the potential vital environmental factors regulating the variations in bacterial and archaeal communities during an *U. prolifera* green tide.

## 2. Materials and Methods

### 2.1. Sampling

**Figure 1:**
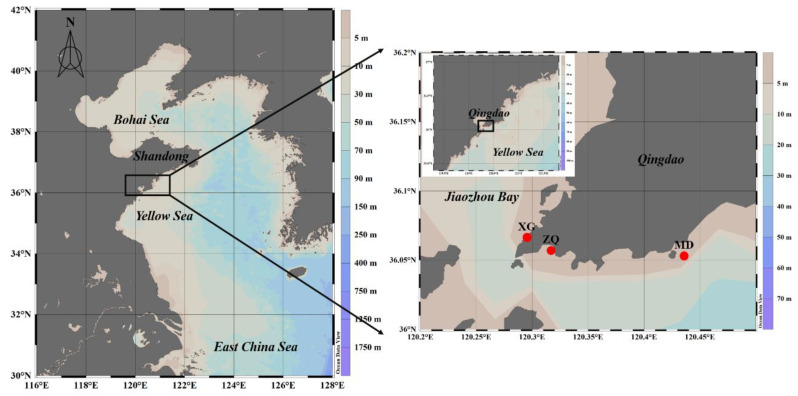
Locations of sampling stations in coastal Qingdao areas during the green tide.

In this study, we chose three stations (XG, ZQ and MD) in coastal Qingdao areas to study the variations in marine bacterial and archaeal communities during an *U. prolifera* green tide (Figure 1). Among three sampling stations, XG (36.07° E, 120.30° N) was less likely impacted by the green tide, whereas ZQ (36.06° E, 120.31° N) and MD (36.05° E, 120.42° N) were greatly influenced by the green tide. Twenty liters of surface seawater at each station was collected from 13 June to 5 September 2019 (Table 1). After collection, the seawater was filtered through an 800-mesh plankton net to remove large zooplankton, then filtered through a 0.22 μm pore-size polycarbonate membrane. The obtained filters were immediately frozen in liquid nitrogen and stored at −80 °C until further molecular analysis.

Temperature, salinity, dissolved oxygen content (DO) and pH were recorded in situ using a YSI proplus multi-parameter water quality analyzer (YSI, Yellow Springs, OH, USA). Chlorophyll *a* (chl*a*) content was determined using the spectrophotometric method after extraction using acetone. To determine the nutrient concentrations, the seawater was filtered through a 0.45 μm pore-size GF/F membrane. Then, concentrations of nitrate (NO_3_^−^), nitrite (NO_2_^−^), ammonium (NH_4_^+^) and phosphate (PO_4_^3−^) were measured with a QuAAtro nutrient auto analyzer (Seal Analytical Ltd., King’s Lynn, UK).

### 2.2. DNA Extraction and Illumina High-Throughput Sequencing

Genomic DNA was extracted with FastDNA Spin kit for Soil (MP Biomedicals, Solon, OH, USA). The obtained DNA was checked through 1% agarose gel electrophoresis analysis, and quantified with NanoDrop 2000 UV-Vis spectrophotometer (Thermo Scientific, Waltham, MA, USA). The hypervariable regions (V3–V4) of bacterial and archaeal 16S rRNA genes were amplified with the primer pairs 338F (5′-ACTC CTA CGG GAG GCA GCA G-3′) and 806R (5′-GGA CTA CHV GGG TWT CTA AT-3′), 524F10extF (5′-TGY CAG CCG CCG CGG TAA-3′) and Arch958RmodR (5′-YCC GGC GTT GAV TCC AAT T-3′), respectively [34,35]. The amplification procedures were as follows: initial denaturation at 95 °C for 3 min, followed by 27 cycles of denaturation at 95 °C for 30 s, annealing at 55 °C for 30 s and extension at 72 °C for 45 s, and a final extension at 72 °C for 10 min. After purification and quantification, the amplicons were pooled in equimolar, then sequenced on Miseq PE300 platform (Illumina, San Diego, CA, USA) at Majorbio Bio-Pharm Technology Co., Ltd. (Shanghai, China). The raw reads were submitted to National Centre for Biotechnology Information (NCBI) Sequence Read Archive (SRA) database under accession numbers PRJNA732997, PRJNA739445, PRJNA741737 and PRJNA742371.

### 2.3. Sequence Processing

Through Fast Length Adjustment of Short reads (FLASH, version 1.2.11, Magoč and Salzberg, Baltimore, MD, USA) [36], raw reads which meet all the following criteria: (i) overlap sequences longer than 10 bp, and (ii) mismatch ratio of overlap greater than 0.2, were merged. The merged sequences were quality-filtered using Quantitative Insights into Microbial Ecology (QIIME, version 1.9.1, Caporaso et al., Boulder, CO, USA) [37]. Briefly, sequences were discarded if they met any of the following criteria: (i) an average quality score below 20, (ii) shorter than 50 bp, and (iii) containing any ambiguous base. Then, operational taxonomic units (OTUs) were clustered with a 97% sequence similarity cutoff using UPARSE (version 7.1, Edgar, CA, USA) [38]. Chimeric sequences were identified and removed at the same time. The predominant sequence in each OTU was chosen as the representative sequence, and annotated with the Silva database (Silva SSU128) using a confidence threshold of 70%.

### 2.4. Statistical Analysis

To illustrate the alpha-diversity of bacterial and archaeal communities, Chao1 index, Shannon index and Good’s coverage were calculated using QIIME. Principal component analysis (PCA) at the OTU level was applied to evaluate the similarities or differences in community structure between the outbreak and decline phases. The top 15 taxa at the family level were compared between the outbreak and decline phases through Wilcoxon rank-sum test. Redundancy analysis (RDA) was employed to explain how environmental factors influenced the bacterial and archaeal communities during the green tide. Correlations of Chao1 index, Shannon index, and relative abundance of dominant taxa with chl*a* content were estimated with SPSS (version 26, IBM Corporation, New York, NY, USA). One-way analysis of variance (ANOVA) was carried out to determine whether there were statistically significant differences among stations or phases.

Co-occurrence network analysis was conducted at the out level during the green tide, the outbreak phase and the decline phase, respectively. OTUs occurring in less than 50% of samples were filtered from total sequences. The network was performed with the package “fdrtool” and “igraph” in R statistical software (version 4.0.4, R Core Team, Vienna, Austria). Only the OTUs with statistically significant values (*p* < 0.01 and *Q* value < 0.05) and the Spearman’s coefficient > |0.6| were put into the further analysis. The co-occurrence network was visualized in Gephi (version 0.9.2, Bastian et al., Paris, France) [39]. The topology of network, including degree, modularity, average path length and average clustering coefficient, were also calculated with Gephi.

## 3. Results

### 3.1. Variations in Environmental Factors during the Green Tide

In this study, a total of 29 surface seawater samples were collected during the green tide. Variations in environmental factors in coastal Qingdao areas during the green tide were shown in Table 2. Over the sampling period, temperature, salinity, pH and DO ranged from 18.60–26.50 °C, 29.28–30.39, 7.58–8.37 and 3.92–12.86 mg/L, respectively. Chl*a* contents ranged from 0.65–4.86 μg/L. NH_4_^+^ concentrations varied from 3.25–28.70 μmol/L, while NO_2_^−^ and NO_3_^−^ concentrations were relatively low with values of 0.04–0.65 μmol/L and 0.59–9.57 μmol/L, respectively. PO_4_^3−^ concentrations varied from 0.10–4.67 μmol/L.

Based on the chl*a* content, all samples were divided into two phases, that is, the outbreak phase (13 June to 22 June 2019) and the decline phase (6 August to 5 September 2019). Temperature (*p* < 0.01), chl*a* content (*p* < 0.01), NO_2_^−^ concentration (*p* < 0.01) and NO_3_^−^ concentration (*p* < 0.01) varied significantly between the outbreak and decline phases.

### 3.2. Variations in Bacterial and Archaeal Richness and Diversity during the Green Tide

In total, 1,590,865 and 1,270,196 high-quality bacterial and archaeal 16S rRNA gene sequences were generated in this study, with an average length of 415 bp and 428 bp, respectively. All high-quality bacterial and archaeal sequences separately yielded 1088 bacterial OTUs and 163 archaeal OTUs. Good’s coverage was greater than 99.51% in all cases, demonstrating that the sequences we obtained sufficiently covered the majority of bacterial and archaeal taxa in this study.

For the bacterial community, Chao1 index and Shannon index were in the range of 539.73–949.26 and 2.31–4.87, respectively (Table 3). Compared with the bacterial community, lower archaeal Chao1 index and lower archaeal Shannon index were observed, ranging from 56.00–123.00 and 1.61–2.95, respectively. The average value of bacterial Chao1 index and the average value of bacterial Shannon index were greater during the decline phase, and the average value of archaeal Chao 1 index and the average value of archaeal Shannon index showed similar trends. Thus, it is speculated that the decline phase is beneficial to the richness and diversity of bacterial and archaeal communities. In addition, we also observed that the diversity of bacterial community differed significantly among stations (*p* < 0.05), whereas the diversity of archaeal community varied markedly between the outbreak and decline phases (*p* < 0.05).

### 3.3. Variations in Bacterial and Archaeal Community Structure during the Green Tide

**Figure 2:**
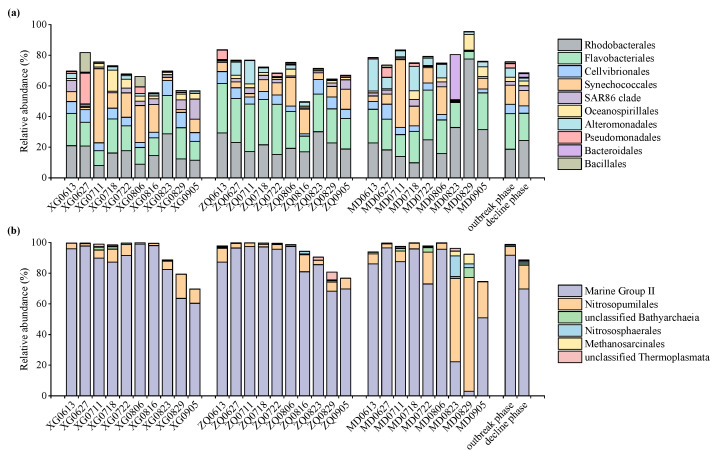
Relative abundance of dominant bacterial (**a**) and archaeal (**b**) taxa at the order level during the green tide.

Thirty bacterial phyla and five archaeal phyla were observed during the green tide. Here, the bacterial and archaeal community composition was analyzed at the order level. *Rhodobacterales* (with a relative abundance ranging from 8.07–77.47%) and *Flavobacteriales* (4.93–32.76%) dominated the bacterial community across all samples (Figure 2a). The relative abundance of *Flavobacteriales* was significantly greater during the outbreak phase compared with the decline phase (*p* < 0.05). *Rhodobacterales* exhibited greater abundance during the decline phase, but not to an obvious extent (*p* > 0.05). *Cellvibrionales* (0.55–9.96%), *Synechococcales* (0.08–48.33%), *SAR86 clade* (0.01–13.11%), *Oceanospirillales* (0.39–13.72%), *Alteromonadales* (0.27–20.81%), *Pseudomonadales* (0.01–20.04%), *Bacteroidales* (0.04–29.39%) and *Bacillales* (0.00–12.84%) also occupied greater proportions in the bacterial community during the green tide. *Cellvibrionales* (*p* > 0.05), *Synechococcales* (*p* > 0.05), *Alteromonadales* (*p* < 0.05), *Pseudomonadales* (*p* > 0.05) and *Bacillales* (*p* > 0.05) were more abundant during the outbreak phase; in contrast, *SAR86 clade* (*p* > 0.05), *Oceanospirillales* (*p* > 0.05) and *Bacteroidales* (*p* > 0.05) exhibited greater abundance during the decline phase.

The predominant archaeal orders comprised of *Marine Group II* (*MGII*, 2.95–98.90%), and to a lesser extent, *Nitrosopumilales* (0.95–74.36%), unclassified *Bathyarchaeia* (0.03–6.31%), *Nitrososphaerales* (0.00–13.57%), *Methanosarcinales* (0.00–6.28%) and unclassified *Thermoplasmata* (0.00–5.04%, Figure 2b). It is noteworthy that *MGII* dominated the archaeal community during the green tide (except for MD0823 and MD0829), and differed markedly between the outbreak and decline phases (*p* < 0.01). Other dominant archaeal taxa, including *Nitrosopumilales*, *Nitrososphaerales*, *Methanosarcinales* and unclassified *Thermoplasmata*, presented greater values during the decline phase, but this was non-significant (*p* > 0.05).

**Figure 3:**
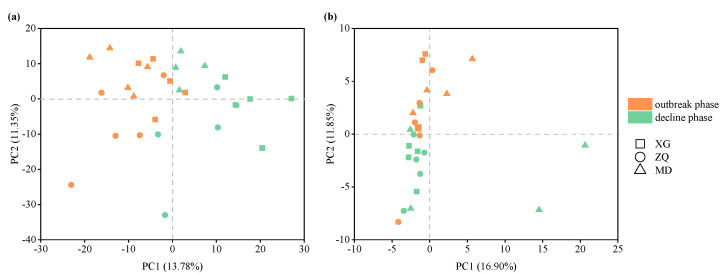
Principal component analysis (PCA) of bacterial (**a**) and archaeal (**b**) communities at the OTU level during the green tide.

**Figure 4:**
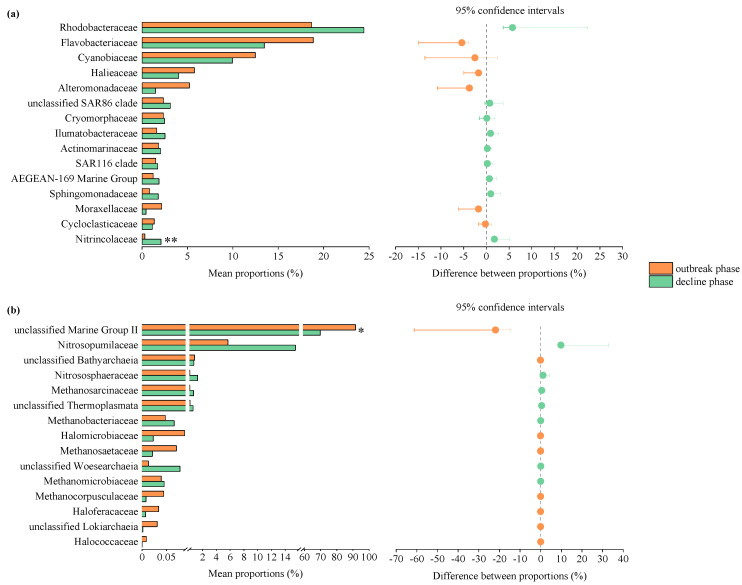
Wilcoxon rank-sum test of the top 15 bacterial (**a**) and archaeal (**b**) taxa at the family level during the green tide. * *p* < 0.05, ** *p* < 0.01.

Clear variations in the bacterial community structure, as well as the archaeal community structure, were observed between the outbreak and decline phases (Figure 3). Based on the analysis of similarities (ANOSIM), both the bacterial and archaeal communities revealed significant differences between the outbreak and decline phases (*p* < 0.01). To identify the differences in community compositions between the outbreak and decline phases, the top 15 taxa at the family level were analyzed using the Wilcoxon rank-sum test. The variations in the bacterial community during the green tide were caused by increases in *Rhodobacteraceae*, unclassified *SAR86 clade*, *Cryomorphaceae*, *Ilumatobacteraceae*, *Actinomarinaceae*, *SAR116 clade*, *AEGEAN-169 Marine Group*, *Sphingomonadaceae* and *Nitrincolaceae*, and decreases in *Flavobacteriaceae*, *Cyanobiaceae*, *Halieaceae*, *Alteromonadaceae*, *Moraxellaceae* and *Cycloclasticaceae* (Figure 4a). Decreases in unclassified *MGII*, unclassified *Bathyarchaeia*, *Halomicrobiaceae*, *Methanosaetaceae*, *Methanocorpusculaceae*, *Haloferacaceae*, unclassified *Lokiarchaeia* and *Halococcaceae*, and increases in *Nitrosopumilaceae*, *Nitrososphaeraceae*, *Methanosarcinaceae*, unclassified *Thermoplasmata*, *Methanobacteriaceae*, unclassified *Woesearchaeia* and *Methanomicrobiaceae* led to the variations in the archaeal community during the green tide (Figure 4b).

### 3.4. Correlation Analysis of Environmental Factors with Bacterial and Archaeal Communities

**Figure 5:**
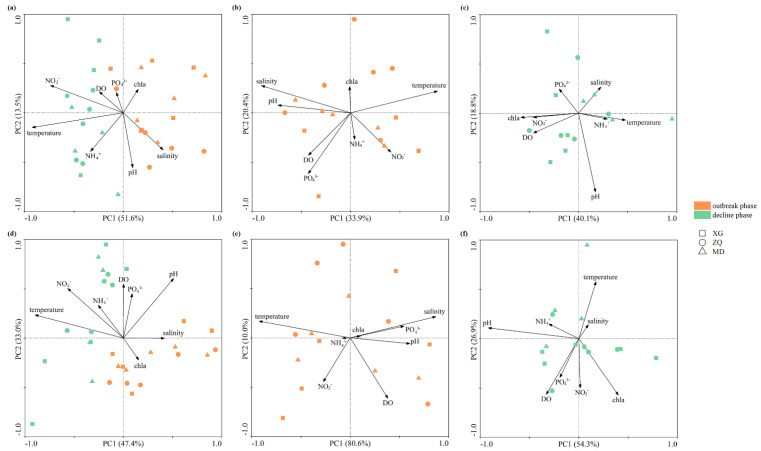
RDA analysis of environmental factors with bacterial community during the green tide (**a**), the outbreak (**b**) and decline (**c**) phase, and with archaeal community during the green tide (**d**), the outbreak (**e**) and decline (**f**) phase.

RDA was employed to explore the correlation of environmental factors with bacterial and archaeal communities. Temperature (*p* < 0.01, *F* = 7.29, 999 Monte Carlo permutations) and NO_2_^−^ concentration (*p* < 0.05, *F* = 2.08, 999 Monte Carlo permutations) contributed most to the variations in bacterial community during the green tide (Figure 5a). The archaeal community during the green tide was strongly associated with temperature (*p* < 0.01, *F* = 8.77, 999 Monte Carlo permutations), pH (*p* < 0.01, *F* = 6.30, 999 Monte Carlo permutations), NO_2_^−^ concentration (*p* < 0.01, *F* = 4.04, 999 Monte Carlo permutations) and salinity (*p* < 0.05, *F* = 2.33, 999 Monte Carlo permutations) (Figure 5d). Meanwhile, it was observed that the bacterial community during the outbreak phase was significantly related to salinity (*p* < 0.01, *F* = 3.44, 999 Monte Carlo permutations) and PO_4_^3−^ concentration (*p* < 0.01, *F* = 2.31, 999 Monte Carlo permutations) (Figure 5b), while temperature (*p* < 0.01, *F* = 16.86, 999 Monte Carlo permutations) exhibited marked correlations with archaeal community during the outbreak phase (Figure 5e). It is noteworthy that during the decline phase, only the archaeal community was found to be significantly impacted by pH (*p* < 0.01, *F* = 5.74, 999 Monte Carlo permutations) (Figure 5f).

**Figure 6:**
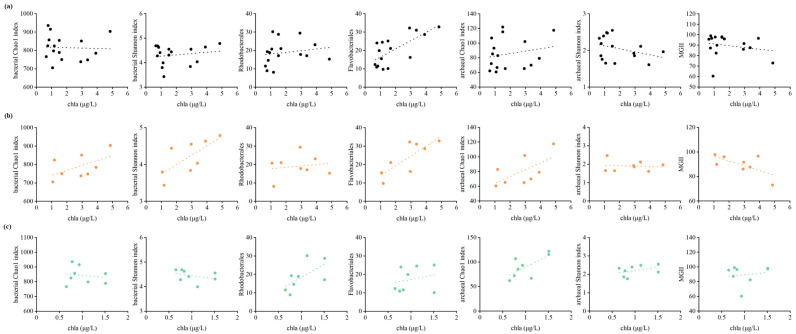
Correlations of Chao1 index, Shannon index, relative abundance of dominant taxa with chl*a* content during the green tide (**a**), the outbreak (**b**) and decline (**c**) phase.

As previously mentioned, chl*a* content is a vital indicator of *U. prolifera* green tide. In order to illustrate how chl*a* content affected the bacterial and archaeal communities, relationships of Chao1 index, Shannon index, relative abundance of dominant taxa with chl*a* content were explored through Spearman correlation analysis (Figure 6, Table 4). It is demonstrated that chl*a* content (*r* = 0.638, *p* < 0.01) plays a crucial role in the relative abundance of *Flavobacteriales* during the green tide. During the outbreak phase, the Shannon index of bacterial community (*r* = 0.857, *p* < 0.01) and the relative abundance of *Flavobacteriales* (*r* = 0.762, *p* < 0.05) was markedly associated with chl*a* content. Nevertheless, chl*a* content had no significant effect on the Chao1 index, Shannon index and relative abundance of *MGII* of archaeal community during any phases (*p* > 0.05).

### 3.5. Microbial Co-Occurrence Network Analysis during the Green Tide

**Figure 7:**
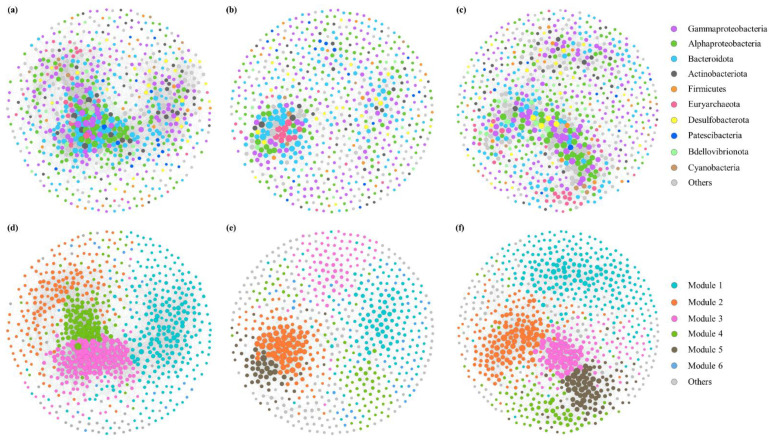
Co-occurrence network colored by taxonomy during the green tide (**a**), the outbreak (**b**) and decline phase (**c**), and colored by modularity during the green tide (**d**), the outbreak (**e**) and decline phase (**f**).

Co-occurrence network was constructed to explore the microbial interactions during the green tide based on the Spearman correlation. Simultaneously, in order to elucidate the distinctions of microbial interactions between phases, we also constructed two separated co-occurrence networks during the outbreak and decline phases. Most of the correlations in the network of green tide were positive, suggesting that mutualism was the main type of interaction among microorganisms during the green tide (Figure 7a). The modularity index of network was 0.464, implying a modular structure during the green tide (Figure 7d). The nodes with relatively more abundance in each module were specific to a particular phase. For example, the majority of OTUs in module 1 and 3 showed greater relative abundance during the outbreak phase, whereas most of OTUs in module 4 had greater relative abundance during the decline phase.

The nodes and edges in the network of the outbreak phase (681 and 3497) were less than those in the network of the decline phase (809 and 6825), suggesting a more complex network during the decline phase (Figure 7b,c). The modularity index of network was 0.577 and 0.585 during the outbreak and the decline phase, respectively, suggesting a more modular structure during the decline phase (Figure 7e,f). In this study, the module with proportion to the whole network greater than 5% was recognized as a major module. During the outbreak phase, the network was attributed to six major modules, named module 1, 2, 3, 4, 5 and 6 accounting for 22.47%, 15.57%, 12.63%, 11.16%, 7.64% and 7.05% of the network, respectively. The network of the decline phase was comprised of five major modules, module 1, 2, 3, 4 and 5, which separately accounted for 31.64%, 21.38%, 12.98%, 11.00% and 10.75% of the network during the decline phase.

In this study, key species should meet the following criteria: (i) have a high degree ranking in the top 20%, and (ii) have a low betweenness centrality ranking in the bottom 20% among the above high degree nodes [40]. During the outbreak and the decline phase, 27 and 32 key species were identified, respectively, which differed between phases. For instance, most of the key species during the outbreak phase belonged to order *Flavobacteriales* within class *Bacteroidia*, whereas order *Rhodobacterales* within class *Alphaproteobacteria* and order *MGII* within class *Thermoplasmata* were observed as key species during the decline phase. Hence, *Flavobacteriales* may play a major part in maintaining the stability of microbial community during the outbreak phase, while *Rhodobacterales* and *MGII* may be crucial for the persistence of the microbial community during the decline phase.

## 4. Discussion

In this study, we explored the variations in marine bacterial and archaeal communities during an *U. prolifera* green tide in coastal Qingdao areas through Illumina high-throughput sequencing analysis. It was concluded that the diversity and structure of bacterial and archaeal communities, as well as the organization and structure of microbial co-occurrence networks, have changed throughout the green tide.

Notable differences on the richness and diversity of both bacterial and archaeal communities were observed between the outbreak and decline phases. In this study, the bacterial and archaeal communities presented greater richness and diversity during the decline phase (Table 3), implying that the decline phase may be beneficial to the bacterial and archaeal richness and diversity. Greater richness and diversity during the decline phase might mean a more complex microbial community at this phase, which was concordant with our results obtained from the microbial co-occurrence network analysis. During the decline phase, decomposing *U. prolifera* released large quantities of organic matter into surrounding seawater. Meanwhile, inorganic nutrient (NH_4_^+^, NO_2_^−^, NO_3_^−^ and PO_4_^3−^) concentrations were also observed to be greater during the decline phase compared with the outbreak phase (Table 2). Large amounts of organic matter and inorganic nutrient concentrations during the decline phase might explain the reason for the greater richness and diversity of bacterial and archaeal communities during this time.

Clear variation in bacterial community structure was observed during the green tide. Overall, *Flavobacteriales* and *Rhodobacterales* dominated the bacterial community during the outbreak and decline phase, respectively (Figure 2a), indicating that they play important roles during the green tide. Due to the rapid response to the enrichment of organic matter, *Flavobacteriales* was regarded as an essential component of the bacterial community during blooms [18,19,41]. Similar to our results, the observations of field studies and incubation experiments in laboratory all exhibited the dominant position of *Flavobacteriales* in the bacterial community during the outbreak phase of green tide [22,24,25]. *Flavobacteirales* were also observed to be more abundant at the late period of the outbreak phase, which may be related to their algicidal activity [42]. *Rhodobacterales* are related to the degradation of organic matter [22], and play a prominent part in various ecological processes, particularly sulfur oxidation and dimethylsulfoniopropionate demethylation [43]. In addition, *Rhodobacterales* have the capability to reduce the nutrient concentrations and hydrogen sulfide contents [43,44,45], which may in turn improve the water quality during the green tide.

It is worth noting that *Alteromonadaceae* exhibited a greater relative abundance during the outbreak phase (Figure 2a), which was in agreement with a previous report on the bacterial community during the bloom caused by *Prorocentrum donghaiense* [46]. Recent studies demonstrated that a certain genus within family *Alteromonadaceae*, such as *Alteromonas*, plays a major part in total bacterial production during blooms, owing to its rapid response to the increasing of organic matter [47]. As one of the first reported algicidal bacteria, *Alteromonas* can secrete algicidal substances to kill certain algae or algal dissolved cells [48,49]. Hence, *Alteromonadaceae*, together with *Flavobacteriales*, might secrete algicidal substances to kill *U. prolifera* and thus dissolve cells directly or indirectly, which may speed up the coming of decline phase [50].

During the *U. prolifera* green tide, the archaeal community was less complex compared with the bacterial community. *MGII* was the predominant archaeal taxon during the green tide (Figure 2b), which is consistent with the results of archaeal community composition during a marine dinoflagellate bloom [19]. *MGII* is the most common archaeal taxon across surface seawater, and it plays a crucial role in carbon and nitrogen cycles, as well as in the attachment and utilization of particulate organic matter in marine ecosystems [51,52,53,54]. Furthermore, we also observed a remarkable increase in the relative abundance of *Nitrosopumilales* during the decline phase (Figure 2b). *Nitrosopumilales*, formerly known as *Marine Group I* (*MGI*), is an important participant in ammonia oxidation and carbon fixation, and also one of the major contributors to marine primary productivity [55,56,57,58]. The lower abundance of *Nitrosopumilus* during the outbreak phase possibly implied a competition for nitrogen nutrients between rapidly growing *U. prolifera* and archaea (ammonia oxidation process) during this phase. Ammonia oxidation, coupled with denitrification, can remove nitrogen pollution [59], thus greater relative abundance of *Nitrosopumilales* during the decline phase may prevent the accumulation of toxic ammonium, reduce the eutrophic level and maintain the nitrogen balance in our studied areas.

As an important indicator of phytoplankton biomass and eutrophication level, chl*a* content was a vital factor affecting the bacterial community during the green tide, mainly through its positive impact on the relative abundance of *Flavobacterales* (*r* = 0.638, *p* < 0.01) during the green tide, and Shannon index (*r* = 0.857, *p* < 0.01) and relative abundance of *Flavobacterales* (*r* = 0.762, *p* < 0.05) during the outbreak phase (Figure 6, Table 4). Nevertheless, chl*a* content did not display any remarkable influences on the archaeal community during any phases, indicating that the bacterial community, rather than the archaeal community, was more sensitive to the outbreak of *U. prolifera*. Temperature may be the principal shaping factor of bacterial and archaeal communities during the green tide, which was shown previously [60,61,62]. For example, Bergen and his colleagues identified that temperature was the major force in structuring the bacterial community during both a diatom bloom in autumn and a mixed phytoplankton bloom in summer [62]; Lucas et al. [63] pointed out that temperature was the main driving factor for the short-term successions of bacterioplankton community during phytoplankton blooms in the German Bight. In this study, salinity also showed the potential to impact the variations in microbial community during the green tide, mainly affecting the archaea. Salinity was one of the most significant environmental factors impacting the distribution of *MGII* archaea [54]. The importance of salinity for the archaeal community may be due to the dominant position of *MGII* in this study.

In addition, complex microbial interactions elucidated by the co-occurrence network also play important roles in regulating the variations in bacterial and archaeal communities during blooms [26], while few studies have paid attention to the microbial interactions during the *U. prolifera* green tide so far. Here, two independent co-occurrence networks during the outbreak and decline phase were established to uncover the differences in microbial interactions between phases, and we found that the organization and structure of microbial co-occurrence networks varied during the green tide. Positive correlations manifest mutualistic interactions, while negative correlations may suggest competition or predation among microorganisms [27,64]. Complex positive or negative correlations among microorganisms were displayed in this study, and mutualistic interactions dominated among the microbial interactions during both the outbreak and decline phases (Figure 7b,c). The proportion of positive correlation during the decline phase (87.44%) was greater than that during the outbreak phase (75.86%), suggesting a greater synergic relationship among microorganisms, which may be in favor of the ecological restoration during the decline phase. The nodes and edges in the network of the outbreak phase were less than those in the network of decline phase, which is in line with the above-mentioned lower microbial richness and diversity during the outbreak phase, indicating that the outbreak of *U. prolifera* may reduce the interactions among microorganisms, as well as the complexity of network structure. In addition, compared with the network of the decline phase, the network of the outbreak phase exhibited lower average degree (4.454 versus 10.523), lower graph density (0.015 versus 0.021), lower clustering coefficient (0.386 versus 0.399) and longer path length (5.082 versus 3.938), indicating that there was a less connected microbial co-occurrence network during the outbreak phase, which may arise from a stronger competition of limited nutrients during this phase [65].

Key species play an exceptionally vital role in maintaining the stability of microbial community [66]. In this study, key species during the outbreak phase were affiliated to *Flavobacteriales*, while *Rhodobacterales* and *MGII* were recognized as the main players during the decline phase, suggesting that *Flavobacteriales*, *Rhodobacterales* and *MGII* may display vital roles during the green tide, and the lack of these organisms may cause disintegration of the structure of microbial co-occurrence networks. As a common bacterial group, *Flavobacteriales* generally degrade organic matter to obtain energy for its rapid growth and also own algicidal activity, which may speed up the arrival of the decline phase [42,50,67]. *Rhodobacterales* and *MGII* are known to participate in diverse biogeochemical cycles, particularly of sulfur, carbon and nitrogen [43,52]. Variations in key species might mean that the microbial potential ecological functions may change during the green tide, and further study is warranted.

## 5. Conclusions

In this study, variations in marine bacterial and archaeal communities during an *U. prolifera* green tide were examined in coastal Qingdao areas. The results revealed that the diversity and structure of bacterial and archaeal communities, as well as the organization and structure of microbial co-occurrence networks, varied during the green tide. The decline phase benefits the bacterial and archaeal richness and diversity. The bacterial community, as well as the archaeal community, exhibited clear variations between the outbreak and decline phases. Moreover, distinct differences in the microbial interactions were also observed between phases, that is, a simpler and less connected microbial co-occurrence network existed during the outbreak phase compared with the decline phase. *Flavobacteriales*, *Rhodobacterales* and *MGII* may be pivotal organisms playing irreplaceable roles during the green tide. In addition, temperature, chlorophyll *a* content and salinity may have an important influence on the variations in bacterial and archaeal community during the green tide.

## Figures and Tables

**Table 1:** Sampling information.

Sampling Date	Sample Name		
	XG	ZQ	MD
13 June 2019	XG0613	ZQ0613	MD0613
27 June 2019	XG0627	ZQ0627	MD0627
11 July 2019	XG0711	ZQ0711	MD0711
18 July 2019	XG0718	ZQ0718	MD0718
22 July 2019	XG0722	ZQ0722	MD0722
8 August 2019	XG0806	ZQ0806	MD0806
16 August 2019	XG0816	ZQ0816	/
23 August 2019	XG0823	ZQ0823	MD0823
29 August 2019	XG0829	ZQ0829	MD0829
5 September 2019	XG0905	ZQ0905	MD0905

**Table 2:** Environmental factors in coastal Qingdao areas during the green tide.

	Temperature (°C)	Salinity	pH	DO (mg/L)	chl*a* (μg/L)	NH_4_^+^ (μmol/L)	NO_2_^−^ (μmol/L)	NO_3_^−^ (μmol/L)	PO_4_^3−^ (μmol/L)
XG0613	18.73	30.15	8.05	7.61	1.67	4.27	0.13	1.53	2.88
XG0627	20.90	29.96	8.05	7.56	1.07	24.35	0.14	1.67	4.31
XG0711	22.80	29.83	7.98	7.31	1.18	5.37	0.08	1.15	0.30
XG0718	23.60	29.78	8.00	6.74	/	23.47	0.17	2.30	0.26
XG0722	24.77	29.49	7.99	7.34	2.96	13.37	0.33	5.02	0.44
XG0806	25.83	29.95	7.58	7.62	0.75	12.66	0.26	2.90	0.61
XG0816	26.10	29.28	7.75	7.48	0.83	18.42	0.65	9.57	0.30
XG0823	26.10	29.66	8.02	10.71	1.51	15.32	0.27	3.70	0.21
XG0829	26.07	29.79	8.37	6.47	/	8.01	0.23	3.08	0.10
XG0905	25.37	29.61	8.09	9.42	0.65	28.70	0.52	6.84	3.92
ZQ0613	20.10	30.08	8.11	9.39	2.92	28.66	0.13	1.76	0.24
ZQ0627	22.00	30.07	8.05	7.24	3.91	18.78	0.11	1.37	0.89
ZQ0711	23.80	30.01	8.04	5.59	3.37	5.69	0.07	0.73	/
ZQ0718	23.70	29.86	8.01	5.08	/	21.13	0.11	1.38	/
ZQ0722	25.90	29.86	8.04	7.94	4.86	5.71	0.08	1.06	/
ZQ0806	26.00	30.39	7.82	7.68	0.78	11.17	0.20	2.09	2.68
ZQ0816	26.20	30.00	8.02	9.10	1.51	10.14	0.21	3.98	0.44
ZQ0823	26.10	30.04	8.11	10.20	1.13	20.74	0.40	5.03	/
ZQ0829	26.50	30.17	8.25	12.17	/	16.01	0.16	1.87	0.27
ZQ0905	25.20	29.88	8.10	12.86	0.93	21.07	0.27	3.56	1.83
MD0613	18.60	30.22	8.11	8.74	/	3.25	0.04	0.59	0.44
MD0627	20.80	30.04	8.13	9.07	/	7.69	0.08	1.07	0.27
MD0711	22.90	29.98	8.13	8.37	/	18.40	0.05	0.73	0.25
MD0718	23.00	29.87	8.05	7.84	/	4.91	0.07	0.97	0.35
MD0722	24.30	29.77	8.05	8.19	/	23.02	0.11	1.93	0.18
MD0806	25.80	29.88	8.01	8.05	/	21.40	0.20	2.66	0.32
MD0823	26.20	29.90	7.99	3.92	/	19.95	0.08	0.99	0.27
MD0829	26.40	29.91	8.21	11.05	/	22.23	0.31	4.76	0.22
MD0905	25.50	29.74	8.08	8.35	/	23.03	0.38	5.45	4.67

/, Missing data.

**Table 3:** Total OTUs, Chao1 index, Shannon index and Good’s coverage of bacterial and archaeal communities during the green tide.

	Bacterial Community				Archaeal Community			
	OTUs	Chao1	Shannon	Coverage	OTUs	Chao1	Shannon	Coverage
XG0613	643	750.36	4.44	0.9965	57	65.27	1.64	0.9995
XG0627	588	705.50	3.80	0.9963	53	60.50	1.65	0.9996
XG0711	659	824.28	3.43	0.9958	82	83.00	2.47	0.9999
XG0718	768	871.21	4.61	0.9963	101	123.00	2.43	0.9996
XG0722	735	851.32	4.55	0.9962	89	102.00	1.85	0.9995
XG0806	671	825.01	4.28	0.9956	57	72.00	1.85	0.9994
XG0816	750	856.65	4.62	0.9961	59	85.25	1.76	0.9994
XG0823	756	854.70	4.31	0.9963	106	115.23	2.55	0.9994
XG0829	787	890.15	4.87	0.9964	89	114.00	2.45	0.9991
XG0905	674	765.88	4.69	0.9967	53	62.00	2.33	0.9996
ZQ0613	642	738.04	3.84	0.6697	64	65.20	1.92	0.9999
ZQ0627	719	784.32	4.63	0.9973	73	79.07	1.61	0.9995
ZQ0711	625	748.04	4.04	0.9959	59	69.91	2.11	0.9994
ZQ0718	813	932.72	4.76	0.9962	83	92.07	2.13	0.9994
ZQ0722	811	903.24	4.79	0.9963	92	117.50	1.96	0.9993
ZQ0806	803	935.37	4.68	0.9959	90	106.87	2.19	0.9991
ZQ0816	712	789.11	4.55	0.9969	102	122.00	2.11	0.9994
ZQ0823	650	797.66	3.99	0.9956	62	66.67	2.49	0.9997
ZQ0829	837	949.26	4.85	0.9962	112	120.57	2.60	0.9994
ZQ0905	762	915.25	4.41	0.9958	76	93.10	2.39	0.9993
MD0613	632	753.15	3.89	0.9967	67	67.75	1.95	0.9999
MD0627	687	781.61	4.45	0.9969	49	56.00	1.61	0.9997
MD0711	646	769.10	3.34	0.9960	70	75.60	2.42	0.9997
MD0718	806	916.57	4.52	0.9963	83	113.00	2.18	0.9991
MD0722	750	830.53	4.23	0.9965	82	84.50	2.37	0.9998
MD0806	792	945.38	4.37	0.9954	86	98.67	2.19	0.9993
MD0823	395	539.73	3.43	0.9968	57	64.50	2.95	0.9998
MD0829	438	631.80	2.31	0.9960	75	80.60	2.36	0.9997
MD0905	617	812.63	3.83	0.9951	83	87.58	2.39	0.9996

**Table 4:** Spearman correlation analysis of Chao1 index, Shannon index, relative abundance of dominant taxa with chl*a* content during the green tide, the outbreak and decline phase.

	Phases	Bacterial Community				Archaeal Community		
		Chao1	Shannon	*Rhodobacterales*	*Flavobacteriales*	Chao1	Shannon	*MGII*
chl*a* content	green tide	−0.212	0.018	0.326	0.638 **	0.209	−0.250	−0.165
	outbreak phase	0.548	0.857 **	−0.048	0.762 *	0.619	0.000	−0.476
	decline phase	0.048	−0.476	0.690	0.333	0.667	0.476	0.143

* *p* < 0.05, ** *p* < 0.01.

## Data Availability

The raw reads were submitted to National Centre for Biotechnology Information (NCBI) Sequence Read Archive (SRA) database under accession numbers PRJNA732997, PRJNA739445, PRJNA741737 and PRJNA742371.
